# Employment status and mental health care use in times of economic contraction: a repeated cross-sectional study in Europe, using a three-level model

**DOI:** 10.1186/s12939-015-0153-3

**Published:** 2015-03-11

**Authors:** Veerle Buffel, Vera van de Straat, Piet Bracke

**Affiliations:** Department of Sociology, Health and Demographic Research group [HeDeRa], Ghent University, Ghent, Belgium

**Keywords:** Macro-economic context and changes, Economic crisis, Mental health care use, Employment status, Gender and age differences, Cross-sectional research design, Eurobarometer

## Abstract

**Introduction:**

Framed within the recent economic crisis, in this study we investigate the medical mental health care use of the unemployed compared with that of the employed in Europe, and whether the relationship between employment status and mental health care use varies across macro-economic conditions. We examine whether the macro-economic context and changes therein are related to mental health care use, via their impact on mental health, or more directly, irrespective of mental health.

**Methods:**

We use data from three waves of the Eurobarometer (2002, 2005/2006, and 2010), which has a repeated cross-sectional and cross-national design. Linear and logistic multilevel regression analyses are performed with mental health, contacting a general practitioner, and contacting a psychiatrist for mental health problems as dependent variables. The multilevel design has three levels (the individual, the period-country, and the country), which allows us to estimate both longitudinal and cross-sectional macro-effects. The macro-economic context and changes therein are assessed using national unemployment rates and growth rates in Gross Domestic Product (GDP).

**Results:**

The mean unemployment rate is negatively related to mental health, although for women, this effect only applies to the employed. Among women, no relationship is found between changes in the macro-economic context and mental health. The unemployment rate, and changes in both the unemployment rate and the real GDP growth rate, are associated with men’s care use, regardless of their mental health, whereas this does not hold for women. In countries with an increase in the unemployment rate, both unemployed and employed men tend to medicalize their problems more by contacting a general practitioner, irrespective of their mental health, while the likelihood of contacting a psychiatrist is lower among employed men.

**Conclusions:**

Our findings stress the importance of taking the macro-economic context and changes therein into account when studying the mental health care use of unemployed people compared with the employed, in particular among men. Moreover, it is important to make the distinction between primary and specialized medical care use, as the impact of macro-economic conditions is dependent on the type of care, which also applies when controlling for mental health.

**Electronic supplementary material:**

The online version of this article (doi:10.1186/s12939-015-0153-3) contains supplementary material, which is available to authorized users.

## Background

The economic crisis has hit Europe since 2008, resulting in rising unemployment rates, worsening of working conditions and losses of income [1,2]. Recent research in some countries, such as Spain and Greece, has shown that the recession has increased the frequency of health problems, especially with regard to mental health [3-6]. In addition, austerity policies might adversely affect health and health care provision [7,8]. Vulnerable groups, such as the unemployed, are found to be the most at risk of deterioration in health and health care access [4,8]. However, also the employed seem to perceive more stress and a reduced mental well-being due to increasing job insecurity and involuntary part-time work [1,2]. The last decades, a trend of flexibilization on the labour-market has already led to a heightened sense of job insecurity [9,10]. This increase in job insecurity may have been further exacerbated by the crisis.

The rise in unemployment, one of the most pressing consequences of the recent economic crisis, makes it particularly important to understand the consequences of unemployment for mental health and its relationship with professional care use. Evidence has consistently shown that unemployment is associated with increased depressive feelings [11,12]. However, the relationship between employment status and care seeking is less straightforward. Some studies have found that the unemployed are less inclined to seek specialized care than the employed [13,14], whereas others have reported greater health care use among the inactive, irrespective of actual (mental) health status [15-17]. In addition, several previous studies use mental health care or psychotropic drug use merely as a proxy for mental health problems [18-20].

There is little recent research that has investigated whether the relationship between employment status and mental health care use varies across macro-economic conditions. Besides, the limited work that has been done on this theme lacks a strong theoretical base [21]. Only in the 1980s, a few studies were published that related aggregated economic conditions to individual help seeking for emotional problems [21,22]. However, these studies did not evaluate recession and major economic changes, because their data was collected in the USA during a period of normal economic fluctuations. The most obvious difference between the context of these American studies and the current European context is that there actually is a recession [23]. The biggest impact has been felt in Spain and Greece, where unemployment rates more than doubled from 2006 to 2011 [24]. Other differences in comparison to these previous studies include a greater awareness of mental health problems [25] and a slight decrease in stigmatization, partly due to anti-stigma campaigns [26]. In many countries, the mental healthcare system has been reformed by a process of deinstitutionalization [27,28] and provisions have increased for mental health care outside of institutions [28].

In this paper, we investigate medical care use for mental health problems by the unemployed compared with the employed in Europe. We assess the impact of the macro-economic context and changes therein on mental health care use, and whether the relationship between employment status and mental health care use varies across these macro-economic conditions. Contacting a general practitioner (GP) or a psychiatrist for emotional or psychological problems is used as an indicator of medical care use for mental health problems. Macro-economic conditions refer to the national economic context as well as changes to this context over three periods (2002, 2005/2006, and 2010). Furthermore, in the current study, attention is also paid to potential age and gender effects.

The most generic way to describe the state of a country’s economy is by using the unemployment rate and the real Gross Domestic Product (GDP) growth rate [1]. The unemployment rate and real GDP growth reflect the economic cycle and thus the economic and labour-market conditions in a country [29-31]. The European Commission also uses these indicators to classify European countries based on the size effect of the recent crisis [1].^a^ Changes in unemployment rates in particular are a relevant measure to capture the economic turmoil and insecurity faced by the population during periods of economic uncertainty, and are close to the everyday experience of individuals [32]. Additionally, the technical definition of a recessionary episode is based on changes in the real GDP growth rate [33].

### Macro-economic context and changes, employment status, and professional care seeking

Relevant theoretical perspectives that combine macro-socioeconomic conditions with help seeking for mental health problems are scarce and date back to the 1980s. We summarize the most important perspectives, supplement them with more recent insights and try to apply them, as among the first to do so, to the relationship between employment status (unemployed versus employed) and both general and specialized mental health care use. Two broad strands of theoretical perspectives can be distinguished. The first suggests there is an indirect relationship between macro-economic conditions and mental health care use via mental health, while the second strand assumes a direct relationship with mental health care use, irrespective of whether there is a relationship between macro-economic conditions and mental health. Therefore, we name the first group “indirect mechanisms” and the second “direct mechanisms”.

*(1) Indirect mechanisms*

The first class of explanations assumes that economic contraction increases the incidence of mental health problems, and consequently mental health care use. Catalano and Dooley [34] refer to these as “provocation explanations”. These perspectives are partly based on the need hypothesis [35], which states that health care use is mainly need based: those with a higher need for care will also use it more. Provocation explanations assume that the relationship between economic conditions and help seeking for emotional problems is indirect, with actual mental health as a mediating factor [22,34]. Stressful conditions that occur more frequently during recession or in a weak economy –such as being unemployed or suffering financial problems– are important risk factors for mental health problems and in turn increase the probability of seeking help [34]. Based on these ideas, we propose the following hypotheses:

**Hypothesis 1:** If the macro-economic context is poor and/or there is economic contraction, mental health will become worse, which will result in correspondingly higher mental health care use.

**Hypothesis 1.a:** Provocation mechanisms may be stronger among the unemployed [34], as they have fewer resources to anticipate and deal with stressful conditions.

However, even the anticipation of stressful conditions, such as becoming unemployed, and economic instability in itself are argued to negatively affect a person’s mental health [22]. The recent economic recession has also led to a worsening of working conditions for those who still have a paid job [1]. In addition, high unemployment rates –and rising unemployment in particular– are the most significant predictors of job insecurity [36,37]. Previous studies have already shown that job insecurity, involuntary part-time work, and temporary contracts are related to a decrease in well-being and mental health status [38-42]. As a result, economic contraction might also be detrimental for the mental health of the employed.

**Hypothesis 1.b:** Because the macro-economic conditions have led to a worsening of working conditions, we can expect –contrary to hypothesis 1.a– that provocation mechanisms will be more pronounced among the employed.

In addition, we have to note that several population surveys have indicated the relationship between mental health status and medical care use is not straightforward. A substantial number of people in need do not report using health services for mental problems, which is termed unmet need [43,44]. In the work of Catalano and Dooley [21,22], the possibility of unmet need is not considered. Nevertheless, economic contraction may increase mental health problems, although this higher need for care may not always directly translate into a higher health care use. Many European countries have responded to recession with austerity policies. This has raised concerns about a possible increase in unmet need [7,45]. Research has already shown reductions in the use of routine and preventive medical care [4,46]. In several countries, recent health reforms have focused on cost containment [45], often leading to higher prescription fees [47,48] and shortages of medicines and supplies [45,47]. The unemployed can be considered as a risk group for unmet need [8,13,14], because they generally perceive more (structural) thresholds to the use of (mental) health care, such as financial barriers [8].

**Hypothesis 1.c:** If the macro-economic context is poor and/or there is economic contraction, mental health will become worse, which results in a higher unmet need for mental health care, in particular among the unemployed.

Moreover, instead of professional care seeking, it is also possible, and in particular among unemployed men [11,49], that alcohol is used as a substitution of mental health care. Alcohol can function as a kind of coping mechanism to handle stress and anxiety [50].

*(2) Direct mechanisms*

The second set of explanations assumes a direct relationship between economic instability and care seeking, regardless of whether there is a (negative) relationship between economic contraction and actual mental health [21,22]. The uncovering mechanism, for example, suggests that during recession –characterized by overstaffed labour-markets and an oversupply of potential employees– atypical behaviour or distress will be tolerated less and labelled easier as deviant and sick, which is assumed to lead to increased mental health care use, regardless of whether the behaviour is new or has previously been treated [22]. In addition, those who have a job may perceive greater job insecurity and will try to prevent illness that might result in job loss. Therefore, economic contraction possibly leads to the anticipation of distress or depression-related complaints, followed by the (asymptomatic) prophylactic or preventive use of mental health facilities [21,22].

**Hypothesis 2:** If the macro-economic context is poor and/or there is economic contraction, mental health care use will increase, regardless of whether there is an increase in mental health problems.

**Hypothesis 2.a:** Uncovering mechanisms and preventive care use are expected to occur more often among the employed.

Some social researchers have mentioned a “medicalization of unemployment” [51,52]. Medicalization is the process whereby non-medical problems are defined and treated as medical problems [53]. Contrary to the dominant biomedical model, which has a need approach (as explained above), the medicalization perspective assumes that medical care use is not always need-driven and highlights the possibility of over-consumption. We have already found evidence for medicalization of unemployment at the individual level: the mental health care use of the unemployed was higher than expected based on their mental health status [17]. In a report about the health effects of the crisis, the Mental Health Commission [54] warned against medicalizing financial, economic and social problems. In times of economic recession in particular, we can expect the process of medicalization of unemployment to be stronger, in response to the greater uncertainty of finding a new job. Individual treatment or medical therapy is often an easy solution, although changing the social circumstances of those affected by the crisis would be more effective and constructive.

**Hypothesis 2.b:** Contrary to hypothesis 2.a, the unemployed in particular will have a higher mental health care use than assumed based on their mental health status, when the economic context is poor and/or there is economic contraction.

In addition, the shift hypothesis [21] assumes that during economic contraction, the type of care that is consulted for emotional problems will change. It has been suggested that economic contraction forces people out of private care and into less costly care in the public sector [4,55]. Also an increased use in generic mental health medication (antipsychotic medicines) is observed [56]. Following this reasoning, the use of more primary care (GPs) and less specialized care (psychiatrists) is expected during a period of recession, given that specialized care is characterized by more thresholds, such as higher fees and a lower supply in most European countries.

**Hypothesis 2.c:** During economic contraction, the likelihood of contacting a GP for mental health problems will increase, while that of psychiatrist consultations decrease, irrespective of the actual mental health status of the individuals.

Finally, we have to remark that this synthesis of perspectives is neither exhaustive nor exclusive, since it is possible that more than one of the mechanisms is at play [21].

### Gender differences

There are several grounds to expect gender differences in care use for emotional problems and the relationship with employment status and macro-socioeconomic conditions. First, women are more likely to label their problems as health related and to accept rather than resist mental health care [21,57].

Second, gender differences in mental health care use have been associated with gender-specific patterns in the pathology of mental disorders: women suffer more from anxiety and depressive disorders, whereas men mainly suffer from impulsive and addictive problems [49]. The latter disorders are associated with a lower demand for care, which might result in the lower use of mental health care by men [58].

Third, the manufacturing and construction sectors suffered the immediate effects of the recession, and these sectors are mainly male dominated [59]. As a result, the absolute number of unemployed men increased more than that of women, especially at the start of the economic crisis [1]. Additionally, individual unemployment may have a stronger negative effect for men. Stigmatization might have a greater impact for unemployed men [60,61] and the financial costs of job loss may also be more pronounced for them, in view of the generally larger share of male earnings in household incomes [62].

Fourth, although there seems to be a relationship between care seeking for emotional problems and economic conditions, it is found to be complex and to vary according to gender [21,22]. Men in particular are at an increased risk of suffering from mental health problems during times of economic adversity [63]. The question is whether men’s care seeking is also more subject to macro-socioeconomic conditions, as Catalano and colleagues [21] found that psychiatric hospital admissions for women vary more quickly than those for men in response to economic change.

### Age effects

Age has been suggested as an important factor in the relationship between employment status and mental health, although evidence is mixed [64]. Unemployment may be more of a problem for middle-aged and older people than for young adults, due to financial and family responsibilities [65]. Conversely, increased unemployment during economic recession has a greater effect on mental health and suicide at younger ages [66]. The majority of young people are eager to enter a vocation, only to discover that few jobs are available, forcing them to accept work for which they are overqualified. Furthermore, a larger proportion of young people have to drop out of school due to their family’s inability to financially support them [3]. In addition, age seems to be directly related to the type of care sought. Research has indicated that younger people find it easier to seek specialized mental health care, whereas older people perceive more socio-cognitive barriers such as stigma, which makes them prefer to use more general care [35].

## Methods

### Sample data

The current study uses data from the Eurobarometer (wave 58.2 in 2002; wave 64.4 in 2005–2006 and wave 73.2 in 2010), which has a repeated cross-sectional survey design. The three waves gathered information from a general population aged 15 and over in member and candidate member countries of the European Union (wave 58.2: 15 countries, wave 64.4: 30 countries and wave 73.2: 28 countries). The basic sample design used in all the countries was a multi-stage, random (probability) sample of individuals within households within an area. Interviews were conducted face-to-face in the national languages. To ensure nationally-representative samples, post-stratification weights are applied according to demographics, using the most recent census data for each country. For more information about the construction of these weights see elsewhere [http://www.gesis.org/eurobarometer-data-service/survey-series/standard-special-eb/weighting-overview/]. Each national sample is representative of the population aged 15 years and above. In line with suggestions from other authors [67], we do not weight the samples according to population size, as the population sizes of the sampled countries are highly heterogeneous. In addition, we have to remark that only for wave 58.2 the response rates per country are available^b^; and not for wave 64.4 and 73.2, which is an important limitation of the Eurobarometer data. We merge the data from East and West Germany, and from Northern Ireland and the rest of the United Kingdom. To operationalize change variables optimally (see the analytical procedure section), we only use information from countries that are present in at least two waves.^c^ As a result, we retain 27 countries, which are presented and specified by survey year in Additional file 1: Table S1. The complete dataset of the three waves contains 32,774 men and 37,978 women.

We use a subsample limited to 23,570 male and 28,646 female respondents of working age (20 to 65 years old). Because no variable contains more than 1.7% missing values, the accumulated percentage of missing values for men is 2.6% (n = 592) and for women 2.2% (n = 632)^d^. These cases are omitted from the sample. As a result, the final sample contains information on 22,978 men and 28,014 women. The number of respondents per country and period are also provided in Additional file 1: Table S1.

### Measurements

#### Mental health care use

Respondents were asked whether they had sought help from a medical professional for a mental health problem in the 12 months preceding the interview. General and specialized care are distinguished, therefore two dummies are constructed: contacting a general practitioner and contacting a psychiatrist (1 = yes; 0 = no).

#### Mental health

The short 5-item version of the Mental Health Inventory (MHI-5), a subscale of the SF-36 Health Survey version 2 [68] measuring depression and anxiety-related complaints, is used as an indicator of mental health care need. The scale ranges from 1 to 5 with high scores pointing to less psychological distress and low scores indicating more psychological distress. If one or two items are missing, mean substitution is applied. The internal reliability of the MHI-5 scale is good (Cronbach’s alpha for men = 0.803; for women = 0.828). There is also existing evidence for the external validity [69] and comparability across countries [70,71].

Employment status contains three categories: unemployed (reference group), employed and non-employed. The non-employed group includes homemakers, students, retired people and those who are unable to work due to illness or disability.

Age is a metric variable and *period* a categorical variable:^e^ 2002, 2005/2006, and 2010, with 2005/2006 used as the reference category. We argue that it is important to take period into account when examining mental health and help-seeking behavior [72]. By including the period variable in the models, we can control for time trends, such as normal economic cycles, trends in mental health care use (e.g. societal processes of medicalization or demedicalization), changes to health, social, and labor-market policies, and changes in healthcare systems (e.g. deinstitutionalization of mental health patients, community-oriented mental health care). In addition, by taking 2005/2006 as the reference period, we are able to compare the situation during the economic crisis (the 2010 period), which began in Europe at the end of 2007 [1], with the situation in the most recent period before the recession (2005/2006).

To control for possible structural thresholds for care seeking, we include some crude indicators of the availability of mental health services, which can also influence help seeking [73]. At the country level, the numbers of GPs and psychiatrists per 10,000 inhabitants are operationalized using information from the OECD 2010 for GPs [74], and the Mental Health Atlas 2005 –or 2011 if information for 2005 was not available– for psychiatrists [75,76]. We also take into account whether or not the country has a gatekeeping system [77]. When there is a gatekeeping system, a patient cannot consult a specialist without first visiting a GP [77]. To consider within-country differences, we control for the *degree of urbanization* using the following categories: large town (reference category), rural area or village, and small or medium-sized town. This can be considered as a proxy for supply [78], because the availability of medical professionals may vary from a large city to a more rural area [79]. In addition, mental health care attitudes may differ by urbanization, with a greater reluctance to seek professional help in rural areas [80].

We also control for *marital status* (married (reference group), divorced, widowed or single) and *educational level*. The respondents were asked at what age they finished full-time education, and the European Commission [81] has provided a standard categorization of the answers: finished at ages through 15 (reference category), finished at ages 16–19, and finished at ages 20 and older; which correspondents roughly to primary, secondary, and tertiary education. In Additional file 2: Table S2 a description of the sample with the individual variables by period and gender is given.

As already mentioned, the *unemployment rate* and *real GDP growth rate* are used as indicators of the macro-economic context, and changes in both are used as proxies for the changing economic context. To calculate these contextual and change variables, we use external data from Eurostat (Labor Force Survey) [82] for the unemployment rates and data from the World Bank^f^ for the GDP growth rates [83], which are shown in Additional file 3: Table S3. Data for the year before the interview year is used, because the respondents were asked whether they had sought professional help in the 12 months preceding the interview and because of the expected time lag^g^. For the context variables, we calculate the *mean unemployment rate* and *the mean real GDP growth rate* over the periods per country. The correlation between the two measurements does not exceed r = 0.4 and the results are also controlled for multicollinearity^h^. The way in which the change variables –*change in the unemployment rate* and *change in the real GDP growth rate*– are operationalized will be explained in the following section, as this is related to the statistical procedure we use.

### Statistical procedure

We use a micro dataset consisting of a series of repeated cross-sectional sample surveys. Respondents are clustered within periods and countries. The Eurobarometer includes information of around 27 European countries, but has only three repeated waves with information about mental health and care use. Like most repeated cross-sectional surveys, we thus face a problem of obtaining an adequate number of higher-level units at the period level [84], since three periods are not enough to include period as an extra level in our multilevel analysis [85]. However, given the cross-national nature of the Eurobarometer, there is a possible solution to this lack of sufficient repeated waves, as has previously been described by Fairbrother [86]: considering the clustering of different waves clustered within countries. National-level time-series cross-sectional data has the advantage that it enables simultaneously modelling cross-sectional (or structural) effects that explain between-country differences, and longitudinal (or change) effects that explain within-country differences over time^i^.

**Figure 1:**
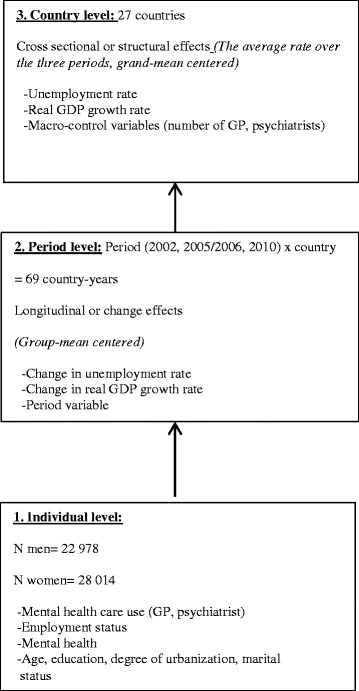
**Presentation of the three-level model, with the number of units and the variables per level.**

In sum, as you can see in Figure 1, respondents, as units of the individual level (level 1), are nested within country-years ranging from 2002 to 2010 at the period level (level 2), which are in turn nested within countries (level 3). Given that not every country participated in every wave (15 countries in 2002 and 27 in 2005/2006, and 2010), we have a multilevel design of 69 different country-years at the period level, and 27 countries at the country level. Figure 1 also specifies, per level, the different variables that will be included in the models.

To include longitudinal (or change) effects at the period level and cross-sectional (or structural) effects of unemployment rate and real GDP growth rate at the country level in the same model, the longitudinal effects are group-mean centered, as described by Fairbrother [86]. Group-mean centering implies that the variables are measured as deviations from the group-mean, in this case the country mean of the unemployment rate and the real GDP growth rate over the three periods. The cross-sectional effects at country level are grand-mean centered: the context variables are thus centered on the overall mean. In this way, the longitudinal effects of the change indicators are orthogonal to the cross-sectional effects. Table 1 presents the descriptive results for these context and change indicators –real GDP growth rate and unemployment rate– separately for men and women per country. Tables 2 and 3 also contain some descriptive results. Table 2 shows the mean scores on mental health and the percentages of GP and psychiatrist consultations for men and women per country and period, while in Table 3, they are given per employment status category.

**Table 1:** **Descriptives: Context and change indicators, real GDP growth rate and unemployment rate, of women and men per country**

	**Real GDP growth rate**				**Unemployment rate of women**				**Unemployment rate of men**			
	**Context Change variable**				**Context Change variable**				**Context Change variable**			
**Country**	**Mean (** $\overline{\mathrm{x}}$ **)**	**2001-** $\overline{\mathrm{x}}$	**2004-** $\overline{\mathrm{x}}$	**2009-** $\overline{\mathrm{x}}$	**Mean (** $\overline{\mathrm{x}}$ **)**	**200 1-** $\overline{\mathrm{x}}$	**2004-** $\overline{\mathrm{x}}$	**2009-** $\overline{\mathrm{x}}$	**Mean (** $\overline{\mathrm{x}}$ **)**	**2001-** $\overline{\mathrm{x}}$	**2004-** $\overline{\mathrm{x}}$	**2009-** $\overline{\mathrm{x}}$
Belgium	0.6	0.3	2.8	−3.2	8.4	−0.9	1.1	−0.3	7.1	−1.2	0.4	0.7
Denmark	−0.6	1.4	3.2	−4.5	5.6	−0.1	0.4	−0.3	5.3	−1.2	−0.2	1.3
Germany	−0.9	2.6	2.1	−4.7	8.4	−0.5	1.7	−1.2	8.8	−1.1	1.8	−0.8
Greece	1.4	2.3	3.6	−5.8	15.2	0.9	1.1	−1.9	7.0	0.2	−0.3	0.0
Spain	1.2	2.8	2.0	−4.8	16.0	−0.8	−1.2	2.1	11.2	−3.7	−2.9	6.5
France	0.6	1.4	2.2	−3.5	8.9	−1.1	0.8	0.3	8.4	−0.5	−0.2	0.6
Ireland	1.2	4.1	3.4	−7.6	5.3	−1.5	−1.3	2.9	8.0	−3.9	−3.2	7.0
Italy	−0.7	2.5	2.3	−4.8	10.6	1.5	−0.1	−1.3	6.7	0.2	−0.3	0.1
Luxembourg	0.5	1.5	4.4	−5.8	5.0	−2.6	1.8	0.9	3.2	−1.6	0.4	1.3
Netherlands	0.1	1.5	1.8	−3.4	4.1	−0.9	1.2	−0.3	3.6	−1.5	1.3	0.1
Portugal	0.2	1.7	1.6	−3.2	7.7	−2.6	0.0	2.6	8.0	−2.8	−0.1	3.0
United Kingdom	0.3	2.4	2.2	−4.6	5.0	−0.6	−0.7	1.4	6.4	−0.9	−1.3	2.1
Austria	0.1	1.3	2.6	−3.9	4.7	−0.5	0.7	−0.1	4.2	−1.1	0.3	0.8
Sweden	0.2	1.4	4.1	−5.4	6.9	−1.3	0.2	1.1	7.4	−1.3	0.2	1.2
Finland	−0.6	3.2	4.5	−7.7	8.7	1.0	0.2	−1.1	8.7	−0.1	0.0	0.2
Republic of Cyprus	1.3		3.0	−3.0	5.8		0.3	−0.3	4.4		−0.9	0.9
Czech Republic	0.1		4.9	−4.9	8.8		1.1	−1.1	6.5		0.6	−0.6
Estonia	−4.1		10.6	−10.6	9.7		−0.6	0.6	13.9		−2.8	2.8
Hungary	−0.9		5.7	−5.7	7.9		−1.8	1.8	8.2		−2.1	2.1
Latvia	−4.7		13.4	−13.4	13.1		−1.1	1.1	16.2		−4.7	4.7
Lithuania	−3.7		11.1	−11.1	10.9		0.4	−0.4	13.8		−3.3	3.3
Malta	−1.7		1.2	−1.2	8.3		0.7	−0.7	6.5		0.0	0.0
Poland	3.9		1.3	−1.3	14.4		5.8	−5.8	13.1		5.3	−5.3
Slovakia	0.0		5.3	−5.3	16.1		3.2	−3.2	14.5		3.0	−3.0
Slovenia	−1.7		6.1	−6.1	6.4		0.6	−0.6	5.9		0.0	0.0
Bulgaria	0.8		5.8	−5.8	9.2		2.5	−2.5	9.7		2.8	−2.8
Romania	1.2		8.0	−8.0	6.1		0.7	−0.7	8.1		0.8	−0.8

Source: Real GDP growth rates from the World Bank, unemployment rates from Eurostat (2001, 2004 & 2009), and own calculations.

**Table 2:** **Descriptives: Mental health and mental health care use of women and men, per country and period**

	**Mental health**												**GP consultations**						**Psychiatrist**					
	**Women**						**Men**						**Women**			**Men**			**Women**			**Men**		
	**2002**		**2005/6**		**2010**		**2002**		**2005/6**		**2010**		**2002**	**2005/6**	**2010**	**2002**	**2005/6**	**2010**	**2002**	**2005/6**	**2010**	**2002**	**2005/6**	**2010**
**Country**	$\overline{\mathrm{x}}$	**SD**	$\overline{\mathrm{x}}$	**SD**	$\overline{\mathrm{x}}$	**SD**	$\overline{\mathrm{x}}$	**SD**	$\overline{\mathrm{x}}$	**SD**	$\overline{\mathrm{x}}$	**SD**	**%**	**%**	**%**	**%**	**%**	**%**	**%**	**%**	**%**	**%**	**%**	**%**
Belgium	3.8	0.8	3.9	0.8	3.8	0.7	3.9	0.8	4.1	0.7	3.8	0.7	8.1	8.2	14.8	4.9	7.0	10.1	1.4	3.2	3.2	2.2	2.9	1.1
Denmark	3.9	0.8	4.1	0.6	3.9	0.6	3.9	0.7	4.1	0.7	4.0	0.5	5.2	12.6	17.0	4.9	11.6	10.6	1.6	1.6	2.4	2.3	0.8	2.0
Germany	3.7	0.8	3.8	0.7	3.8	0.6	3.8	0.7	3.9	0.6	3.8	0.7	7.2	10.8	10.3	4.2	7.6	8.4	0.8	2.0	1.7	0.6	1.2	1.5
Greece	3.6	0.8	3.5	0.8	3.4	0.7	3.9	0.7	3.7	0.7	3.5	0.6	1.7	8.3	5.0	1.9	5.3	2.7	1.2	2.8	0.8	0.3	0.7	0.3
Spain	3.8	0.8	3.8	0.8	3.7	0.7	4.0	0.7	3.9	0.7	3.8	0.6	3.6	4.9	14.7	2.5	3.7	8.2	1.6	1.0	2.9	0.3	1.9	1.7
France	3.6	0.9	3.8	0.8	3.7	0.7	3.8	0.8	4.0	0.7	4.0	0.7	9.2	9.0	13.8	6.7	9.0	10.4	2.8	4.2	1.9	2.1	1.8	2.0
Ireland	3.8	0.7	3.9	0.6	3.9	0.6	4.0	0.7	4.0	0.7	4.0	0.6	6.9	16.0	13.7	3.7	7.3	6.8	0.8	0.7	0.7	1.1	0.3	0.9
Italy	3.4	0.8	3.4	0.7	3.4	0.6	3.6	0.7	3.7	0.7	3.6	0.6	1.0	8.3	10.2	1.8	7.4	11.1	1.5	0.6	0.5	0.8	0.9	0.1
Luxembourg	3.7	0.8	4.0	0.7	3.8	0.7	3.9	0.7	4.1	0.6	4.0	0.6	4.3	15.1	9.5	2.9	16.3	8.0	3.5	3.2	2.0	1.9	2.8	3.7
Netherlands	4.0	0.7	3.9	0.8	3.9	0.7	4.0	0.7	4.2	0.6	4.1	0.6	6.3	10.0	11.9	7.0	5.5	9.1	2.0	2.5	3.4	2.2	3.0	3.0
Portugal	3.5	0.9	3.6	0.8	3.7	0.7	4.0	0.7	3.9	0.7	3.8	0.6	10.0	14.5	18.5	3.2	6.9	3.7	3.7	6.3	2.6	1.2	2.0	2.3
United Kingdom	3.6	0.8	3.9	0.7	3.7	0.8	3.7	0.8	4.0	0.6	3.9	0.7	11.1	12.6	17.8	9.4	9.3	9.1	1.2	1.2	1.5	2.4	1.2	1.3
Austria	3.8	0.8	3.8	0.7	3.7	0.7	3.9	0.7	4.2	0.7	3.7	0.7	5.5	13.3	12.7	2.9	9.1	10.7	0.8	2.3	0.9	0.3	0.5	0.5
Sweden	3.9	0.7	4.0	0.7	4.0	0.6	4.1	0.7	4.2	0.6	4.1	0.6	7.6	10.2	11.4	2.3	5.2	6.9	2.7	2.8	1.5	1.7	1.0	2.2
Finland	4.2	0.6	4.1	0.6	4.0	0.6	4.1	0.6	3.9	0.7	4.0	0.6	3.8	5.4	7.4	2.2	4.6	6.6	2.1	2.5	3.0	1.9	3.0	2.2
Republic of Cyprus			3.5	0.8	3.5	0.8			3.9	0.7	3.8	0.7		4.9	6.0		1.2	4.9		1.3	1.5		0.6	2.7
Czech Republic			3.8	0.7	3.8	0.7			3.9	0.7	3.7	0.7		9.3	9.2		7.2	8.2		2.5	1.2		2.4	1.3
Estonia			3.7	0.8	3.6	0.8			3.9	0.8	3.8	0.7		14.4	14.8		10.2	9.5		3.7	3.3		2.7	1.9
Hungary			3.7	0.9	3.6	0.8			3.8	0.8	3.7	0.7		10.1	9.7		6.2	6.3		4.0	3.7		4.0	1.4
Latvia			3.6	0.8	3.5	0.7			3.9	0.8	3.8	0.7		7.5	13.6		5.0	7.4		1.3	0.6		0.7	0.6
Lithuania			3.5	0.8	3.5	0.7			3.6	0.8	3.5	0.7		12.6	14.6		8.8	10.1		3.2	2.6		1.9	2.8
Malta			3.7	0.7	3.6	0.7			3.8	0.7	3.6	0.7		7.2	11.5		6.3	10.2		2.4	1.4		1.8	2.3
Poland			3.7	0.9	3.7	0.8			3.9	0.6	3.8	0.6		7.8	8.2		6.6	4.3		3.3	2.1		2.3	1.0
Slovakia			3.8	0.7	3.7	0.7			3.8	0.8	3.7	0.7		13.1	17.0		10.4	14.6		1.3	1.2		1.6	0.7
Slovenia			3.7	0.7	3.8	0.6			3.9	0.7	3.9	0.6		9.0	5.8		5.0	4.5		1.7	2.4		1.8	2.6
Bulgaria			3.6	0.8	3.6	0.7			3.9	0.6	3.8	0.6		12.6	5.6		9.1	1.7		1.9	0.5		0.5	0.1
Romania			3.6	0.8	3.5	0.7			3.8	0.7	3.6	0.7		24.7	25.4		15.9	24.5		0.5	0.8		0.9	0.5

Source: Eurobarometer 58.2 (2002), 64.4 (2005/2006) and 73.2 (2010).

**Table 3:** **Descriptives: Gender differences in mental health and mental health care use by employment status and period**

		**Mental health (1–5)**					**GP consultations**			**Psychiatrist consultations**		
		**women**		**men**			**women**	**men**		**women**	**men**	
		$\overline{\mathrm{x}}$	**SD**	$\overline{\mathrm{x}}$	**SD**	**sig.** ^**a**^	**%**	**%**	**sig.** ^**b**^	**%**	**%**	**sig.** ^**b**^
2002	Employed	3.8	0.8	3.9	0.7	***	5.5	3.5	***	1.4	1.1	
	Unemployed	3.6	0.9	3.6	0.8		10.5	7.3		2.1	2.9	
	Non-employed	3.7	0.8	3.9	0.8	***	7.0	5.3	*	2.2	2.0	
2005/2006	Employed	3.9	0.7	4.0	0.6	***	8.9	6.1	***	1.5	1.1	*
	Unemployed	3.6	0.8	3.7	0.8		13.5	9.0	**	3.5	1.6	*
	Non-employed	3.6	0.8	3.8	0.8	***	13.2	12.5		3.2	3.6	
2010	Employed	3.8	0.7	3.9	0.6	***	10.8	7.6	***	1.1	0.8	*
	Unemployed	3.5	0.8	3.5	0.8		15.1	10.2	***	3.4	3.0	*
	Non-employed	3.6	0.7	3.8	0.7	***	14.1	12.9		2.5	2.9	

*p < .050 **p < .010 ***p < .001; N individual women = 28014 & men = 22978.

^(a)^Difference between men’s and women’s mean tested via Anova-test.

^(b)^Difference between men’s and women’s proportion tested via pairwise Chi^2^-test.

Source: Eurobarometer 58.2 (2002), 64.4 (2005/2006) and 73.2 (2010).

The actual analyses to test our hypotheses consist of two parts: First, our primary assumption at the individual level is tested, specifically whether unemployment is related to worse mental health compared with being employed (Model 1) and how this varies by age (Model 2). Subsequently, we assess the basic proposition of the first strand of theoretical perspectives –which assumes an indirect relationship between mental health care use and macro-economic conditions via actual mental health– and in this regard we compare the employed with the unemployed. Therefore, we briefly look at the relationship between employment status and mental health and how this relationship is moderated by the macro-economic context and changes therein (Model 3). In the last model we also take the period variable into account (Model 4). Accordingly, a three-level multiple regression analysis is performed, with mental health status as the dependent variable, controlled for other important determinants of mental health (education, age, and marital status) (Table 4).

**Table 4:** **Mental health regressed on employment status, age, and economic context and change variables**

	**Men**								**Women**							
	**Model 1**		**Model 2** ^**a**^		**Model 3**		**Model 4**		**Model 1**		**Model 2** ^**a**^		**Model 3** ^**a**^		**Model 4** ^**a**^	
	**b**	**sig.**	**b**	**sig.**	**b**	**sig.**	**b**	**sig.**	**b**	**sig.**	**b**	**sig.**	**b**	**sig.**	**b**	**sig.**
Intercept	3.629	***	3.620	***	3.632	***	3.693	***	3.371	***	3.364	***	3.391	***	3.601	***
**(1) Individual variables**																
Age^b^	−0.002	*	−0.007	***	−0.002	*	−0.002	*	−0.004	***	−0.010	***	−0.004	***	−0.004	***
Employment status (ref. unemployed)																
Employed	0.272	***	0.281	***	0.268	***	0.269	***	0.157	***	0.184	***	0.159	***	0.159	***
Non-employed	0.177	***	0.175	***	0.174	***	0.175	***	0.093	***	0.117	***	0.095	***	0.095	***
Employed × age^b^			0.004	***							0.007	***				
**(2) Period variables**																
Change in real GDP growth rate^c^					0.009	*	0.001						−0.006		0.003	
Change in unemployment^c^					0.007		0.002						0.003		0.004	
Period (ref. 2005/2006)																
2002							−0.081	**							−0.085	***
2010							−0.105	*							−0.068	*
**(3) Context variables**																
Mean real GDP growth rate^b^					0.009		0.013						−0.017		0.014	
Mean unemployment rate^b^					−0.016	*	−0.016	*					−0.006		−0.014	
Employed × mean unemployment rate^b^					-		-						−0.007	*	−0.006	*
**Variance**																
(3) Country	0.018	*	0.019	**	0.017	**	0.018	**	0.005	*	0.004		0.006		0.015	
(2) Period	0.010	**	0.009	**	0.006	**	0.005	*	0.146	***	0.147	***	0.143	***	0.009	
(1) Individual	0.458	***	0.457	***	0.458	***	0.458	***	0.542	***	0.542	***	0.542	***	0.542	
ρ^d^	0.058		0.058		0.048		0.048		0.218		0.218		0.216		0.042	
**DiC**	47299777		47284117		47297345		47296805		63057254		63037902		63060348		63056399	

*p < .050 **p < .010 ***p < .001; N individual women = 28014 & men = 22978; N period (xcountry) = 69; N country = 27.

Models controlled for education and marital status.

^a^Controlled for interaction effects with the non-employed (model 2 men & women: non-employed × age; model 3–4 women: non-employed × mean unemployment rate).

^b^Variable is grand-mean centered (abstraction of mean of all respondents).

^c^Variable is group-mean centered (abstraction of mean of the group).

^d^Variance at both higher levels: 3 and 2 (country + period) = (σ^2^_country_ + σ^2^_period_)/(σ^2^_country_ + σ^2^_period_ + σ^2^_individual_).

Source: Eurobarometer 58.2 (2002), 64.4 (2005/2006), and 73.2 (2010).

In the second part, we use three-level logistic regression analysis with GP (Table 5) and psychiatrist consultations (Table 6) for mental health problems as the dependent variables. In order to shed light on some mediating paths, we present five models: (1) a baseline model with age, employment status, the control variables (degree of urbanization, education and marital status) and the macro-economic variables at the country level (context variables) and at period level (change variables); (2) a model adjusted for cross-level interaction effects^j^ of employment status with the economic context and change variables; (3) a model controlling for the period variable; and (4) subsequently, we assess to what extent the effects of the macro-economic context and changes therein on mental health care use change when mental health is taken into account; and whether there is also a direct effect of the macro-economic conditions on mental health care use irrespective of mental health. Finally, (5) in the last model, the interaction effects between age and employment status are introduced.

**Table 5:** **General practitioner consultations regressed on employment status, age, mental health, and economic context and change variables**

	**Men**										**Women**									
	**Model 1**		**Model 2** ^**a**^		**Model 3** ^**a**^		**Model 4** ^**a**^		**Model 5** ^**a**^		**Model 1**		**Model 2** ^**a**^		**Model 3**		**Model 4**		**Model 5** ^**a**^	
	**OR**	**sig.**	**OR**	**sig.**	**OR**	**sig.**	**OR**	**sig.**	**OR**	**sig.**	**OR**	**sig.**	**OR**	**sig.**	**OR**	**sig.**	**OR**	**sig.**	**OR**	**sig.**
Intercept	0.304	***	0.313	***	0.349	***	0.251	***	0.256	***	0.380	***	0.389	***	0.390	***	0.374	***	0.369	***
**(1) Individual variables**																				
Age^b^	1.009	***	1.009	***	1.009	***	1.008	***	1.001		1.007	***	1.007	***	1.007	***	1.005	***	1.010	***
Employment status (ref. unemployed)																				
Employed	0.827	***	0.806	***	0.802	***	0.975		0.976		0.786	***	0.784	***	0.790	***	0.873	***	0.865	***
Non-employed	1.050		1.026		1.016		1.143	*	1.142	*	0.888	**	0.889	***	0.893	**	0.938		0.933	
Mental health^b^							0.582	***	0.582	***							0.611	***	0.611	***
Employed × age^b^									1.008	*									0.995	
**(2) Period variables**																				
Change in real GDP growth rate^c^	0.994		0.994		0.997		1.004		1.006		0.986	*	0.985	**	1.002		1.001		1.005	
Change in unemployment^c^	1.031	*	1.031	*	1.024	*	1.021	*	1.021	*	1.031		1.027		1.010		1.010		1.014	
Period (ref. 2005/2006)																				
2002					0.708	*	0.668	***	0.671	***					0.728	***	0.466	***	0.474	***
2010					1.022		1.058		1.078						1.118	*	1.082		1.122	
**(3) Context variables**																				
Mean real GDP growth rate^b^	0.978		0.981		0.983		0.991		0.990		0.990		0.977		0.987		0.993		0.992	
Mean unemployment rate^b^	1.007		0.979		0.970		0.973		0.964		0.983		0.987		0.982		0.980		0.980	
Employed × mean unemployment rate			1.031	*	1.032	*	1.029	*	1.028	*			1.023		-		-		-	
**Variance**																				
(3) Country	0.072		0.087		0.154		0.175		0.197		0.066		0.063		0.121		0.176		0.167	
(2) Period	0.224		0.212		0.085		0.089		0.082		0.196		0.203		0.075		0.079		0.081	
VPC^d^	0.141		0.142		0.117		0.127		0.134		0.126		0.128		0.098		0.123		0.121	
**DiC**	11424972		11424728		11420274		10605999		10605990		18183328		18176035		18179319		16974150		16973980	

*p < .050 **p < .010 ***p < .001; N individual women = 28014 & men = 22978; N period (xcountry) = 69; N country = 27.

Odds ratio’s (OR) are y-standardized; Models controlled for education, marital status and degree of urbanization.

^a^Controlled for the interaction effects with the non-employed (model 2–5 men; model 2 women: non-employed × mean unemployment rate; model 5 men & women: non-employed × age).

^b^Variable is grand-mean centered (abstraction of mean of all respondents).

^c^Variable is group-mean centered (abstraction of mean of the group).

^d^Variance at both higher levels: 3 and 2 (country + period) = (σ^2^_country_ + σ^2^_period_)/(σ^2^_country_ + σ^2^_period_ + 3,29).

Source: Eurobarometer 58.2 (2002), 64.4 (2005/2006), and 73.2 (2010).

**Table 6:** **Psychiatrist consultations regressed on employment status, age, mental health, and economic context and change variables**

	**Men**										**Women**									
	**Model 1**		**Model 2** ^**a**^		**Model 3** ^**a**^		**Model 4** ^**a**^		**Model 5** ^**a**^		**Model 1**		**Model 2** ^**a**^		**Model 3**		**Model 4**		**Model 5** ^**a**^	
	**OR**	**sig.**	**OR**	**sig.**	**OR**	**sig.**	**OR**	**sig.**	**OR**	**sig.**	**OR**	**sig.**	**OR**	**sig.**	**OR**	**sig.**	**OR**	**sig.**	**OR**	**sig.**
Intercept	0.132	***	0.126	***	0.139	***	0.094	***	0.088	***	0.173	***	0.168	***	0.183	***	0.148	***	0.155	***
**(1) Individual variables**																				
Age^b^	1.008	**	1.008	**	1.008	**	1.006	*	0.992		1.003		1.003		1.003		1.001		0.997	
Employment status (ref. unemployed)																				
Employed	0.642	***	0.653	***	0.652	***	0.807	*	0.855		0.638	***	0.647	***	0.637	***	0.751	***	0.759	***
Non-employed	1.021		1.048		1.042		1.166		1.220	*	0.934		0.947		0.933		1.017		1.024	
Mental health^b^							0.491	***	0.489	***							0.516	***	0.516	***
Employed × age^b^									1.016	*									1.005	
**(2) Period variables**																				
Change in real GDP growth rate^c^	1.010		0.988		0.987		0.983		0.987		1.006		0.996		0.997		0.998		0.999	
Change in unemployment^c^	1.030		1.029		1.023		1.022		1.026		1.024		1.025		1.011		1.011		1.012	
Employed × change GDP growth rate			1.035	*	1.034	*	1.041	*	1.043	*			1.013		-		-		-	
Period (ref. 2005)																				
2002					0.916		0.839		0.865						0.850	*	0.490	***	0.494	***
2010					0.966		0.930		0.973						0.879	*	0.876		0.892	
**(3) Context variables**																				
Mean real GDP growth rate^b^	0.955		0.972		0.968		0.971		0.966		0.984		0.985		0.987		0.996		0.991	
Mean unemployment rate^b^	0.973		0.976		0.972		0.957		0.955		0.995		0.994		0.995		0.989		0.985	
**Variance**																				
(3) Country	0.211		0.219		0.215		0.435		0.430		0.230		0.219		0.223		0.326		0.319	
(2) Period	0.027		0.019		0.017		0.024		0.025		0.016		0.029		0.028		0.015		0.016	
VPCd	0.116		0.116		0.114		0.202		0.201		0.120		0.121		0.122		0.159		0.156	
**DiC**	3455473		3452717		3454947		3052591		3052627		5383282		5385256		5381756		4816708		4819491	

*p < .050 **p < .010 ***p < .001; N individual women = 28014 & men = 22978; N period (xcountry) = 69; N country = 27.

The odds ratio’s (OR) are y-standardized; Models controlled for education, marital status and degree of urbanization.

^a^Controlled for the interaction effects with the non-employed (model 2–5 men & model 2 women: non-employed × change GDP growth rate; model 5 men & women: non-employed × age).

^b^Variable is grand-mean centered (abstraction of mean of all respondents).

^c^Variable is group-mean centered (abstraction of mean of the group).

^d^Variance at both higher levels: 3 and 2 (country + period) = (σ^2^_country_ + σ^2^_period_)/(σ^2^_country_ + σ^2^_period_ + 3,29).

Source: Eurobarometer 58.2 (2002), 64.4 (2005/2006), and 73.2 (2010).

All models are estimated in the statistical software package MLwiN using Markov Chain Monte Carlo (MCMC) estimation procedures, as this approach has been shown to be robust, particularly when including cross-level interactions [85]. We only consider random intercept models, as the random slopes are not significant. All the analyses are gender differentiated and the metric independent variables (age, mental health, mean unemployment rate, and mean real GDP growth rate) are grand-mean centered to make interaction effects easier to interpret [87]. To make the odds ratios (ORs) comparable across the nested models, we use y-standardisation^k^ as recommended by Mood [88]. By doing this, we partly take unobserved heterogeneity into account.

## Results

### Descriptive results

First, we briefly discuss some descriptive results. In Table 1, which is a synthesis of the macro-economic context and change variables, it is notable that the change in the real GDP growth rate is positive for each country in the first two periods, whereas it decreases in 2009 in every country. This is a clear reflection of the economic crisis. This is a clear reflection of the economic crisis. With regard to changes in the unemployment rate, unemployment increases among men in the 2009–2010 period in the majority of countries, particularly in Spain, Ireland, Portugal, Latvia, and Lithuania.

The following table (Table 2) shows, as was also found in our previous study [57], that there are large cross-national differences in the use of mental health care, while the differences in mental health are smaller. In general, there is no clear increase or decrease in the level of mental health and in mental health care use between the three periods, as the differences between the periods seem to be largely country specific.

The last table (Table 3) with descriptive results shows mental health and mental health care use by employment status and gender, and whether the differences between men and women are significant (using Anova-tests for the metric variables and Chi^2^-tests for the categorical). For each period, unemployed men and women have poorer mental health, especially compared with the employed. Employed and non-employed men’s mental health is significantly better than that of employed and non-employed women, while this is not the case for unemployed men’s mental health.

When the different employment statuses are compared for women, those who were unemployed were most likely to have contacted a GP or a psychiatrist for mental health problems in each period (with the exception of psychiatrist consultations in 2002), while for unemployed men this was only the case for 2002. The percentages of women –both employed and unemployed– who contacted a GP and a psychiatrist, are significantly higher than those of men in at least two of the three periods.

### Results of the three-level regression analyses

From the variance decomposition of the null model (not shown) we notice that there is relatively little variance in mental health at the higher levels (between-years within countries at level 2 and between-countries at level 3): 5.5% of women’s and 5.9% of men’s mental health are influenced by the country and the period in which they are surveyed (ρ country + period = (σ^2^country + σ^2^period)/(σ^2^country + σ^2^period + σ^2^individual). The variance in care use at the higher levels (Variance Partition Coefficient country + period = (σ^2^country + σ^2^period)/(σ^2^country + σ^2^period + 3.29) is markedly higher, particularly in GP consultations (women: 12.2%; men: 11.6%; psychiatrist consultations respectively 6.3% and 7.1%). This could be a first indication that mental health care use is not just need based.

#### The relationships between employment status and mental health by age, macro-economic context, and changes therein

We start with the basic relationship between individual employment status and mental health (Table 4). As expected, for both women and men the association between being employed and mental health is positive (Model 1: b_women_ = 0.157; b_men_ = 0.272). This relationship changes with age: the mental health gap between the employed and the unemployed is slightly larger among the older respondents (Model 2: b_women_ = 0.007; b_men_ = 0.004).

Next, we are interested in the initial assumption of the first set of theoretical perspectives, which assumes an indirect relationship between macro-economic conditions and mental health care use, via actual mental health. Therefore, we first test whether there is a relation between mental health and the macro-economic context and changes therein (Model 3). In countries with an increase in real GDP growth rate, men’s mental health is slightly better (b = 0.009). However, after controlling for the period variable, this effect is no longer significant (Model 4). We also find that in countries with a high mean unemployment rate, men’s mental health is slightly worse (b = 0.016), irrespective of their individual employment status and the period of study. The first part of hypothesis 1 is thus confirmed for men. Among women, the relationship between the mean unemployment rate and mental health is only found for the employed (b = −0.007; Model 3). This relation also remains significant after controlling for period (Model 4). In addition, in Model 4 we observe that the mean mental health of men and women of working age in 2002 (b_women_ = −0.085; b_men_ = −0.081) and 2010 (b_women_ = −0.068; b_men_ = −0.105) is significantly worse than in 2005/2006.

#### The relationship between employment status and mental health care use by age, macro-economic context, and changes therein

We now examine how individual employment status and the macro-economic context and changes therein are related to mental health care use, for which we turn to Table 5 (GPs) and Table 6 (psychiatrists). To find out whether there is an indirect (Hypothesis 1) or direct (Hypothesis 2) relationship between the macro-economic conditions and professional care use, we start with the baseline models in which the context and change variables, the individual employment status and the control variables (age, education, marital status and degree of urbanization) are included. Men’s likelihood of contacting a GP for mental health problems is higher in countries with an increase in the unemployment rate (OR = 1.031). Women in countries with a decrease in the GDP growth rate are also more likely to contact a GP (OR = 1/0.986). With regard to psychiatrist consultations, we do not find macro-economic effects in the baseline model. At the individual level, the unemployed men and women are significantly more likely to contact a GP (OR _men_ = 1/0.827; OR _women_ = 1/0.786) and a psychiatrist (OR = 1/0.642; OR = 1/0.638) compared to the employed.

Furthermore, interaction effects with employment status are added to test whether the relationship with mental health care use varies across the macro-economic context and changes therein, and thus whether the direct or the indirect mechanisms are more pronounced among the unemployed (Hypothesis 1.a or 2.b) or the employed (Hypothesis 1.b or 2.a). Model 2 of Table 5 shows a positive association between mean unemployment rate and GP consultations for men, but only among the employed: the likelihood of employed men to contact a GP for mental health problems is higher in countries with a higher unemployment rate (OR = 1.031). With regard to men’s psychiatrist consultations (Model 2, Table 6), we see that in countries with a decline in the GDP growth rate, employed men are less likely to contact a psychiatrist (OR = 1/1.035) compared to those in countries with an increase in the GDP growth rate. The relation between an increase in unemployment rate and a higher likelihood of contacting a GP among men, as well as, the relation between a decrease in the real GDP growth rate and the higher likelihood of contacting a GP among women (Models 2 in Table 5) do not vary significantly across employment status.

In the third model, the period variable is taken into account. As a result, women’s relation between change in real GDP growth rate and GP consultations is no longer significant (Model 3, Table 5).

To see whether the relationships between the macro-economic conditions and mental health care use are mediated by mental health (indirect mechanism), or only partly and that they also remain significant regardless of mental health (direct mechanism), mental health status is introduced in Model 4. Among men, the relation between change in unemployment rate and GP consultations only slightly attenuates and remains significant. The interaction effect between change in real GDP growth rate and employment status on men’s psychiatrist consultations even appears to be slightly stronger after taking mental health into account (OR = 1.034 Model 3; OR = 1.041 Model 4). Men’s mental health care use, thus is to some extent directly associated with the macro-economic change and contextual variables, as we find some significant effects after taking mental health status into account; while for women, the main effects of the mean unemployment rate and real GDP growth rate, and changes in both, are not significantly related to mental health care use, after controlling for the period variable and mental health.

By adding the mental health status (Model 4), we can also assess whether the higher mental health care use by the unemployed can be ascribed to their worse mental health status at the individual level (as found in Table 4), which would be in line with the need hypothesis. If the mental health care use of the unemployed remains significantly higher after controlling for actual mental health, this would be an indication of the medicalization theory. The results are primarily in line with the latter, with the exception of GP consultations for unemployed men (Model 4, Table 5).

In addition, there are some interesting period and age effects. In 2002 and 2010, women (OR respectively 0.850 and 0.879; Model 3 Table 6) were less likely to consult a psychiatrist than in 2005/2006. Otherwise, with regard to GP consultations for emotional problems, we observe that in 2010, women were significantly more likely to contact a GP than in 2005/2006 (OR = 1.118; Model 3 Table 5). This can mainly be ascribed to a higher need for care in 2010, as the period effect here is no longer significant after taking mental health into account (Model 4). By contrast, the likelihood of contacting a GP for mental health problems was lower for women (OR = 0.728) and men (OR = 0.708) in 2002 than in 2005/2006, also after controlling for mental health (Model 4).

Only for men, some interaction effects of age with employment status are found. If men’s age is higher than the mean (around 43 years old, see Additional file 2: Table S2), employed men (OR_age*employed_ = 1.008) are more likely to visit a GP for mental health problems compared with the unemployed of the same age, irrespective of their mental health. However, when their age is lower than the mean age, employed men are less likely to visit a GP than the unemployed are at the same age. With regard to men’s psychiatrist consultations, the results show that: the older men are, the smaller the difference in psychiatrist consultations between the employed and the unemployed (OR_age*employed_ = 1.016).

## Discussion

In this study, we have assessed whether the relationship between employment status and mental health care use is contingent on the economic climate; and whether the macro-economic context and changes therein are related to mental health care use, via their impact on mental health, or more directly, irrespective of mental health. Our study reveals some important findings.

First, we have briefly examined the relation between macro-economic conditions and mental health. Some results seem to support the basic assumption of the first set of perspectives, which we named indirect mechanisms. Among both unemployed and employed men, mental health is worse in countries with a high mean unemployment rate than in countries with a lower one. Possible explanations are that the unemployed are more pessimistic regarding future prospects, as their chances of re-employment are lower, and that the employed perceive greater job insecurity, a higher work load in shrinking sectors [54], and work intensification (more work pressure and a higher work speed) [2,89], which are risk factors for worse mental health.

For women, the negative relationship between a high unemployment rate and mental health is only applicable to the employed. Thus, in countries with a high unemployment rate, the mental health gap between unemployed and employed women is smaller, as the negative impact is stronger for the employed. A possible explanation can be found in the social norm theory [90,91]. The social norm effect of unemployment assumes that the employed suffer the most from a high unemployment rate, through increasing job insecurity, feelings of guilt, and higher workloads, whereas for those who are unemployed, any social norm effect mitigates the negative effects of unemployment [90]. In this context, unemployment may be perceived more as a structural problem than a personal failure, which can reduce the associated stigma.

The results have also shown that in countries with a decrease in the GDP growth rate, men’s mental health is slightly worse compared to that of men in countries with an increase in their GDP growth rate (but only when period is not taking into account). This may be an indication of a negative effect of the economic recession on men’s mental health, irrespective of their own employment status. This confirms the results of some single-country studies, performed in countries that were highly affected by the crisis, such as Spain and Greece [3,5,6]. Among women, hardly any evidence is found for increased mental health problems in a situation of economic contraction, characterized by a strong increase in the unemployment rate and/or decrease in the real GDP growth rate. This is in line with the results presented by Eurofound, based on the EQLS data [92], which report that mental wellbeing remained fairly stable in Europe during the economic crisis, with the exception of only a few countries. A possible explanation for the rather small effect of economic contraction on men’s mental health and no effect on women’s mental health could be that there are also (mental) health gains associated with economic contraction, which might counter the expected negative impact of the recession [31,93]. However, these positive health effects of the recession were especially related to health behavior (e.g. more physical activity, less alcohol consumption) [31]. An alternative explanation could be that 2010 is a little too late to capture the acute short-term effect or the “shock panic reaction” just after the start of the crisis, whereas on the other hand, it might be too early for evaluating long-term effects on mental health.

Subsequently, the relation with mental health care use was explored. Among men, we found that the relations between macro-economic conditions and GP consultations for mental health problems could only partly be ascribed to the actual mental health status, and the relation with psychiatrist consultations even became slightly stronger. These findings are in line with the second set of perspectives, which assumes that there is a direct relationship between macro-economic conditions and mental health care seeking (Hypothesis 2).

On the one hand, we find that in countries with a high increase in unemployment, general mental health care is used more often by both the employed and the unemployed men, irrespective of actual mental health status. This suggests that the medicalization process is stronger in the countries that were hit hardest by the crisis in terms of unemployment rates. Despite the fact that in times of economic contraction unemployment should be seen as a structural problem, it is also a more desperate situation that possibly is still treated first and foremost as a personal problem. Even among those who have a job, a slight increase in GP consultations is found, which might be explained by increased job insecurity [1]. Our previous research [17] has indeed shown that job insecurity can be medicalized. In addition, in countries with a high mean unemployment rate, employed men are more inclined to contact a GP for mental health problems. As this relationship applies only to employees, it seems to be in line with the uncovering hypothesis and/or the (asymptomatic) prophylactic use of mental health facilities [21,22] (Hypothesis 2.a). Moreover, the medicalization of increased job insecurity could also be a possible explanation here.

On the other hand, with regard to men’s psychiatrist consultations we find that in countries with a decline in the GDP growth rate, the employed are less likely to contact a psychiatrist, regardless of their mental health. This result, in combination with the increase in GP consultations appears to be an indication of the shift hypothesis. An alternative or additional explanation, which could explain why this finding only applies to working people, may be that the employed may avoid specialized care use for fear of being labelled as sick, acquiring a treatment stigma [94], and consequently losing their job [46].

Among women, we found that in countries with a decline in GDP growth rate, there is an increase in GP-consultations. However, this relation could be ascribed to period effects and the actual mental health status. This finding in combination with the period effects, suggests an indirect relationship between macro-socioeconomic conditions and general mental health care (GP consultations) among women (Hypothesis 1). Women were more likely to contact a GP for mental health problems in 2010 than in 2005/2006, which could be ascribed to their worse mental health in 2010. The provocation explanation should link the period of general economic contraction (2010) to more depressive and anxiety symptoms that are, in turn, linked to more general help seeking [22]. This would also be consistent with the need hypothesis [35]. This conclusion does not seem to extend to specialized mental health care, as the likeliness of contacting a psychiatrist was lower in 2010. This could indicate an increase in unmet need for specialized care, or a shift to more accessible general health care in times of economic contraction.

In addition, we want to pay attention to some interesting results at the individual level. Consistent with previous research [11,12], the unemployed do have a worse mental health than the employed, and consequently a higher need for professional care. As expected, the negative relationship between unemployment and mental health is stronger for the middle and older age groups. The observed higher mental health care use by the unemployed, however, cannot be ascribed solely to their poorer mental health, with the exception of unemployed men’s GP consultations, which are mainly need based. Unemployed men and women use more specialized medical care (psychiatrists), and unemployed women also use more general medical care (GP) for mental health problems than would be expected based on their need for care. These findings are in line with some previous studies [15-17], and support the “medicalization of unemployment” hypothesis: using medical care not merely in response to mental health problems, but as a way to cope with unemployment [17]. Stress and other negative emotional feelings resulting from unemployment could lead to isolated non-specific symptoms, which are reclassified as diseases for which medical treatment is sought [51]. Based on our results, we cannot simply say that this medicalization of unemployment is more pronounced among the younger or the older respondents. We find that at an older age the differences in the use of psychiatrist consultations decrease between the employed and the unemployed, regardless of their mental health, but only for men. Further research using data covering a wider time span could be useful here, in order to explore whether this age effect is instead a hidden cohort effect.

Finally, some limitations of the study should be addressed. First, the Eurobarometer data has some problems with regard to temporal order. The main independent variable–employment status–indicates the situation of the respondents at the time of the interview. However, the items concerning professional care seeking refer to the twelve months preceding the interview, and the period of reference for experiencing depressive feelings is the preceding four weeks. As a result, we cannot differentiate between processes of causation and those of reverse causation. This is normal for most cross-sectional studies [14,78], but it contributes to blurring the time ordering of the main variables. Accordingly, we attempted to control for possible selection biases and problems of endogeneity in various way. Reverse causality is a concern if individuals with poorer health are more likely to be unemployed. As we separate those who were inactive due to illness or disability from the category of the unemployed, we reduce this possible reverse causality. The models are also estimated taking the country’s unemployment rate into account. By doing this, we control for potential between-country differences in selection bias related to between-country variation in the proportion of the unemployed. However, with the available data, we cannot give a definite answer regarding the direction of the relationships.

Second, we are unable to consider income due to limitations of the dataset used. Financial means are an important enabling resource with regard to professional care use [95,96]. After controlling for mental health, low-income groups are found to use fewer mental health services, in particular specialized care. Nevertheless, the indicators for education and employment situation may at least partially replace income effects [78].

Third, the information about mental health is self-reported and only takes depression and anxiety-related complaints into account. This is a relatively limited operationalization of mental health status and need–a description strictly in terms of “mental illness” and not in terms of “inability to function”. In addition, the expression of stress and mental health problems differs between men and women, and accordingly it would be better to also include indicators of impulsive and addictive behaviour, such as alcohol abuse [49]. Similar to mood and anxiety disorders, this type of behavior can also be related to unemployment [11] and is found to be negatively associated with health care use [58,97].

## Conclusions

In sum, although the evidence for the medicalization theory is quite convincing at the individual level –the unemployed have a higher medical care use than expected based on their need for mental health care [17]– this is less the case when the macro-economic context and changes therein are also considered. A shortcoming of the medicalization perspective, which is revealed throughout this study, is that it does not take the type of care system (primary versus specialized medical care) into account. As we find different trends in primary and specialized care use for mental health problems when paying attention to the impact of macro-economic conditions this seems to be very important. The results suggest an increase in GP consultations for mental health problems during poor economic conditions, whereas we find a decrease in psychiatrist consultations during economic contraction, irrespective of mental health status.

Moreover, although macro-economic conditions seem to be directly associated with mental health and professional care seeking of men, they possibly also have indirect consequences for wellbeing and mental health care use through their effect on public policies [98]. Therefore, in a future study we aim to examine the role of a country’s austerity policies in response to the crisis, given that active labour market programs, strong social safety nets, and mental health prevention campaigns seem to mitigate the negative mental health effects of recession [32,99].

Finally, as the current study helps to understand how the mental health care use of the unemployed versus the employed is related to the economic context and changes therein, further research needs to explore the role of specific characteristics of a country’s health care system and social policies. In addition, although we focus here on the unemployed versus the employed, we recognize that within these two groups there are also important differences. For the unemployed these include, for example the duration and the reason of job loss [100], and for the employed, intrinsic and extrinsic job characteristics [40,41]. These differences might also be related to mental health and mental health care use. Moreover, dependent on social class and socio-economic position, some individuals more will be more vulnerable to individual unemployment and to the impact of the macro-economic context and changes therein than others, which may also have consequences for their mental health and medical care use. Accordingly, further research that goes beyond the dichotomy of employed versus unemployed is certainly required.

## Endnotes

^a^However, we recognize that there are other indicators of the macro-economic context, which are also appropriate as macroeconomic proxies. For example, the notification rate of plant closings and mass layoffs, as was used by Gerdtham and Johannesson [101], is also useful as a good indicator of the labour-market condition. Unfortunately, this information is not widely available [30], which is quite problematic given the number of countries and periods included in our study.

^b^For the response rates per country of the Eurobarometer wave 58.2, see page 13: [http://ec.europa.eu/health/ph_determinants/life_style/mental_eurobaro.pdf]

^c^Therefore Croatia (2005), Cyprus (TCC) (2005), Turkey (2005) and Iceland (2010) are left out of the analyses.

^d^In the first column of Additional file 2: Table S2, the percentages of missing values per variable are shown.

^e^These specifications for age and period resulted in the best model fit.

^f^For the real GDP growth rates we had to rely on the data of the World bank [83], as Eurostat has no information for 2001 [82]. The real GDP growth rates for 2004 and 2009 of the World bank [83] are similar to the numbers of Eurostat [82].

^g^Using external data of the year before the data collection also resulted in the best model fit.

^h^The absence of multicollinearity is not an assumption for logistic regression analysis. However, as we also perform linear regression analysis with mental health as a dependent variable, we have to take a look at the assumption of “absence of multicollinearity”. Therefore, we have computed (in SPSS) the Variance Inflation Factor, VIF. For any variable in the model the VIF was a lot lower than 10, which means that there is no problem of multicollinearity.

^i^An important assumption related to this method is that these models presuppose that social change happens within countries over time [86]: time trends are nested within each survey each time. Given the limited number of available country-years containing information about professional care seeking for mental health problems, reliably estimating the assumption that country-years are nested within countries by comparing the model fit to that of the alternative model is not warranted [84]. Therefore we have to assume the nesting of country-years within countries. For this paper, however, notwithstanding that there is a global financial crisis, not every country was affected by or responded to the crisis in the same way [32], which partially supports this assumption.

^j^If the interaction effects are not significant, they are excluded from the analysis to enhance interpretability and to obtain a more parsimonious model.

^k^This means that the coefficient is divided by the sum of the standard deviation of the predicted logit, and the assumed standard deviation of the error term (which is always the square root of 3.29) [88].

## Additional files

Additional file 1: Table S1. Percentage of cases with missing values on the variables and the final sample size of women and men per country per period. Source: Eurobarometer wave 58.2 (2002), wave 64.4 (2005/2006) and wave 73.2 (2010). Additional file 2: Table S2. Description of the sample: the individual variables by period and gender. and their % missing values. Source: Eurobarometer wave 58.2 (2002), wave 64.4 (2005/2006) and wave 73.2 (2010). Additional file 3: Table S3. Description of the external data: Real GDP growth rate and unemployment rate of women and men by period and country. Source: World Bank for real GDP growth rates; Eurostat for the unemployment rates (2001, 2004 and 2009).

## Abbreviations

GP: General practitioner
GDP: Gross domestic product
MHI-5: Mental health inventory scale with 5 items
MCMC: Markov Chain Monte Carlo
OR: Odds ratio
SE: Standard error
OECD: Organisation for Economic Cooperation and Development
EQLS: European Quality of Life Survey

## References

[1] Eurofound. Impact of the crisis on working conditions in Europe. European Foundation for the Improvement of Living and Working Conditions: Dublin, Ireland;2013. http://eurofound.europa.eu/sites/default/files/ef_files/docs/ewco/tn1212025s/tn1212025s.pdf 2013

[2] Green F (2014). Work intensification, insecurity and well-being in Britain’s workplaces: recent trends [IOE Research Briefing N°98].

[3] Economou M, Madianos M, Peppou LE, Patelakis A, Stefanis CN (2013). Major depression in the era of economic crisis: a replication of a cross-sectional study across greece. J Affect Disorders..

[4] Kentikelenis A, Karanikolos M, Papanicolas I, Basu S, McKee M, Stuckler D (2011). Health effects of financial crisis: omens of a Greek tragedy. Lancet..

[5] Gili M, Roca M, Basu S, McKee M, Stuckler D (2013). The mental health risks of economic crisis in Spain: evidence from primary care centres, 2006 and 2010. Eur J Public Health..

[6] Madianos M, Economou M, Alexiou T, Stefanis C (2011). Depression and economic hardship across Greece in 2008 and 2009: two cross-sectional surveys nationwide. Soc Psych Psych Epid..

[7] McKee M, Karanikolos M, Belcher P, Stuckler D (2012). Austerity: a failed experiment on the people of Europe. Clin Med..

[8] Kyriopoulos II, Zavras D, Skroumpelos A, Mylona K, Athanasakis K, Kyriopoulos J (2014). Barriers in access to healthcare services for chronic patients in times of austerity: an empirical approach in Greece. Int J Equity Health..

[9] Sparks K, Faragher B, Cooper CL (2001). Well-being and occupational health in the 21st century workplace. J Occup Organ Psych..

[10] Hartley J, Jacobson D, Klandermans B, van Vuuren T (1991). Job insecurity: coping with jobs at risk.

[11] Bartley M (1994). Unemployment and Ill health-understanding the relationship. J Epidemiol Commun H..

[12] Paul KI, Moser K (2009). Unemployment impairs mental health: meta-analyses. J Vocat Behav..

[13] Alonso J, Codony M, Kovess V, Angermeyer MC, Katz SJ, Haro JM (2007). Population level of unmet need for mental healthcare in Europe. Brit J Psychiat..

[14] Gouwy A, Christiaens W, Bracke P (2008). Mental health services use in the general Belgian population: estimating the impact of mental health and social determinants. Archive of Public Health..

[15] Bijl RV, Ravelli A (2000). Psychiatric morbidity, service use, and need for care in the general population: results of the Netherlands Mental Health Survey and Incidence Study. Am J Public Health..

[16] Yuen P, Balarajan R (1989). Unemployment and patterns of consultation with the general-practitioner. Brit Med J..

[17] Buffel V, Dereuddre R, Bracke P. Medicalisation of the uncertainty? An empirical study of the relationships between unemployment or job insecurity, professional care seeking, and the consumption of antidepressants. Eur Sociol Rev. 2015; doi: 10.1093/esr/jcv004

[18] Kuhn A, Lalive R, Zweimüller R. The Public Health Costs of Unemployment. Université de Lausanne, Faculté des HEC, DEEP, http://EconPapers.repec.org/RePEc:lau:crdeep:07.08 2007

[19] Morris JK, Cook DG (1991). A critical-review of the effect of factory closures on health. Brit J Ind Med..

[20] Schmitz H (2011). Why are the unemployed in worse health? The causal effect of unemployment on health. Labour Econ..

[21] Catalano RA, Dooley D, Jackson RL (1985). Economic antecedents of help seeking-reformulation of time-series tests. J Health Soc Behav..

[22] Dooley D, Catalano R (1984). Why the economy predicts help-seeking-a test of competing explanations. J Health Soc Behav..

[23] Suhrcke M, Stuckler D (2012). Will the recession be bad for our health?. It depends. Soc Sci Med..

[24] OECD. Employment and Labour Market Statistics: Labour force statistics by sex and age http://dx.doi.org/10.1787/unemp-table2131-en

[25] Schomerus G, Schwahn C, Holzinger A, Corrigan PW, Grabe HJ, Carta MG (2012). Evolution of public attitudes about mental illness: a systematic review and meta-analysis. Acta Psychiat Scand..

[26] Paykel ES, Hart D, Priest RG (1998). Changes in public attitudes to depression during the Defeat Depression Campaign. Brit J Psychiat..

[27] Rutz W (2001). Mental health in Europe: problems, advances and challenges. Acta Psychiat Scand..

[28] Nome S, Holsten F (2011). A prospective longitudinal study of utilization of a psychiatric hospital in Hordaland County, Norway, from 1985 to 2003. Nord J Psychiat..

[29] Economou A, Nikolaou A, Theodossiou I (2008). Socioeconomic status and health-care utilization: a study of the effects of low income, unemployment and hours of work on the demand for health care in the European Union. Health services management research: an official journal of the Association of University Programs in Health Administration / HSMC, AUPHA..

[30] Gerdtham UG, Ruhm CJ (2006). Deaths rise in good economic times: evidence from the OECD. Econ Hum Biol..

[31] Ruhm CJ (2000). Are recessions good for your health?. Q J Econ..

[32] Stuckler D, Basu S, Suhrcke M, Coutts A, McKee M (2009). The public health effect of economic crises and alternative policy responses in Europe: an empirical analysis. Lancet..

[33] Directorate general for Employment, Social Affairs and Equal opportunities. European Commision: Publications Office of the European union: Luxembourg http://ec.europa.eu/social/home.jsp?langId=en 2010

[34] Catalano R, Dooley CD (1977). Economic predictors of depressed mood and stressful life events in a metropolitan community. J Health Soc Behav..

[35] McAlpine DD, Boyer CA, Avison WR, McLeod JD, Pescosolido BA (2007). Sociological traditions in the study of mental health services utilization. Mental health, social mirror.

[36] Dixon JC, Fullerton AS, Robertson DL (2013). Cross-national differences in workers’ perceived Job, labour market, and employment insecurity in Europe: empirical tests and theoretical extensions. Eur Sociol Rev..

[37] Esser I, Olsen KM (2012). Perceived job quality: autonomy and Job security within a multi-level framework. Eur Sociol Rev..

[38] De Witte H (1999). Job insecurity and psychological well-being: review of the literature and exploration of some unresolved issues. European Journal of Work and Organizational Psychology..

[39] Ferrie JE, Shipley MJ, Stansfeld SA, Marmot MG (2002). Effects of chronic job insecurity and change in job security on self reported health, minor psychiatric morbidity, physiological measures, and health related behaviours in British civil servants: the Whitehall II study. J Epidemiol Commun H..

[40] Virtanen M, Kivimaki M, Ferrie JE, Elovainio M, Honkonen T, Pentti J (2008). Temporary employment and antidepressant medication: a register linkage study. J Psychiatr Res..

[41] De Moortel D, Vandenheede H, Muntaner C, Vanroelen C (2014). Structural and intermediary determinants of social inequalities in the mental well-being of European workers: a relational approach. BMC Public Health..

[42] Bambra C, Lunau T, Van der Wel KA, Eikemo TA, Dragano N (2014). Work, health, and welfare: the association between working conditions, welfare states, and self-reported general health in Europe. Int J Health Serv..

[43] Alonso J, Angermeyer MC, Bernert S, Bruffaerts R, Brugha IS, Bryson H (2004). Use of mental health services in Europe: results from the European Study of the Epidemiology of Mental Disorders (ESEMeD) project. Acta Psychiat Scand..

[44] Bijl RV, de Graaf R, Hiripi E, Kessler RC, Kohn R, Offord DR (2003). The prevalence of treated and untreated mental disorders in five countries. Health Affair..

[45] Stuckler D, McKee M (2012). There is an alternative: public health professionals must not remain silent at a time of financial crisis. Eur J Public Health..

[46] Gene-Badia J, Gallo P, Hernandez-Quevedo C, Garcia-Armesto S (2012). Spanish health care cuts: penny wise and pound foolish?. Health Policy..

[47] Karanikolos M, Mladovsky P, Cylus J, Thomson S, Basu S, Stuckler D (2013). Financial crisis, austerity, and health in Europe. Lancet..

[48] Bettio F, Corsi M, D’Ippoliti C, Lyberaki A, Lodovici MS, Verashchagina A. The impact of the economic crisis on the situation of women and men and on gender equality policies. Luxembourg, Belgium: Synthesis Report. European Commission Directorate-General for Justice; 2012

[49] Vesga-Lopez O, Schneier FR, Wang S, Heimberg RG, Liu SM, Hasin DS (2008). Gender differences in generalized anxiety disorder: results from the national epidemiologic survey on alcohol and related conditions (NESARC). J Clin Psychiat..

[50] Riska E, Ettorre E (1999). Mental distress-gender aspects of symptoms and coping. Acta Oncol..

[51] Holmqvist M (2009). Medicalization of unemployment: individualizing social issues as personal problems in the Swedish welfare state. Work Employ Soc..

[52] Miles I (1987). Some observations on unemployment and health research. Soc Sci Med..

[53] Conrad P (1992). Medicalization and social-control. Annu Rev Sociol..

[54] Lynch KTD. The Human Cost: an overview of the evidence on economic adversity and mental health and recommendations for action. Mental Health Commission: Synthesis Report; 2011

[55] Karanikolos M, Rechel B, Stuckler D, McKee M (2013). Financial crisis, austerity, and health in Europe Reply. Lancet..

[56] Leopold C, Zhang F, Mantel-Teeuwisse AK, Vogler S, Valkova S, Ross-Degnan D (2014). Impact of pharmaceutical policy interventions on utilization of antipsychotic medicines in Finland and Portugal in times of economic recession: interrupted time series analyses. Int J Equity Health..

[57] Buffel V, Van de Velde S, Bracke P (2014). Professional care seeking for mental health problems among women and men in Europe: the role of socioeconomic, family-related and mental health status factors in explaining gender differences. Soc Psych Psych Epid..

[58] Rhodes AE, Goering PN, To T, Williams JI (2002). Gender and outpatient mental health service use. Soc Sci Med..

[59] Rubery J, Rafferty A (2013). Women and recession revisited. Work Employ Soc..

[60] Forret ML, Sullivan SE, Mainiero LA (2010). Gender role differences in reactions to unemployment: exploring psychological mobility and boundaryless careers. J Organ Behav..

[61] Kulik L (2000). Jobless men and women: a comparative analysis of job search intensity, attitudes toward unemployment, and related responses. J Occup Organ Psych..

[62] Wang JL, Smailes E, Sareen J, Fick GH, Schmitz N, Patten SB (2010). The prevalence of mental disorders in the working population over the period of global economic crisis. Can J Psychiat..

[63] Wahlbeck K, McDaid D (2012). Actions to alleviate the mental health impact of the economic crisis. World Psychiatry..

[64] Goldman-Mellor SJ, Xaxton KB, Catalano RC (2010). Economic contraction and mental health, a review of the evidence, 1990–2009. Int J Mental Health..

[65] Breslin FC, Mustard C (2003). Factors influencing the impact of unemployment on mental health among young and older adults in a longitudinal, population-based survey. Scand J Work Env Hea..

[66] Uutela A (2010). Economic crisis and mental health. Curr Opin Psychiatr..

[67] Frohlich N, Carriere KC, Potvin L, Black C (2001). Assessing socioeconomic effects on different sized populations: To weight or not to weight?. J Epidemiol Commun H..

[68] Ware JE, Sherbourne CD (1992). The Mos 36-item short-form health survey (Sf-36).1. Conceptual-framework and item selection. Med Care.

[69] Mchorney CA, Ware JE, Raczek AE (1993). The Mos 36-item short-form health survey (Sf-36) 2. Psychometric and clinical-tests of validity in measuring physical and mental-health constructs. Med Care.

[70] Lehto-Järnstedt U, Aromaa A (2003). Mental health measurement in comprehensive national health surveys.

[71] Wagner AK, Gandek B, Aaronson NK, Acquadro C, Alonso J, Apolone G (1998). Cross-cultural comparisons of the content of SF-36 translations across 10 countries: Results from the IQOLA project. J Clin Epidemiol..

[72] George LK (2014). Taking time seriously: a call to action in mental health research. J Health Soc Behav..

[73] Koopmans GT, Donker MCH, Rutten FHH (2005). Common mental disorders and use of general health services: a review of the literature on population-based studies. Acta Psychiat Scand..

[74] OECD. Health Resources, Doctors http://data.oecd.org/healthres/doctors.htm

[75] WHO (2011). Mental health atlass 2011.

[76] WHO (2005). Mental health atlas 2005.

[77] Xavier A, Lipszyc B, Sail E, Przywara B. Joint Report on Health Systems. Directorate-General for Economic and Financial Affairs: European Commission; 2010.

[78] Bracke PF, Colman E, Symoens SAA, Van Praag L. Divorce, divorce rates, and professional care seeking for mental health problems in Europe: a cross-sectional population-based study. BMC Public Health 2010, 10: doi:10.1186/1471-2458-10-224 10.1186/1471-2458-10-224 PMC2879244 20429904

[79] Saxena S, Thornicroft G, Knapp M, Whiteford H (2007). Global Mental Health 2-Resources for mental health: scarcity, inequity, and inefficiency. Lancet..

[80] Hoyt DR, Conger RD, Valde JG, Weihs K (1997). Psychological distress and help seeking in rural America. Am J Commun Psychol..

[81] Eurobarometer. Technical Report. European Commission http://ec.europa.eu/public_opinion/archives/eb/eb48/48techspec.pdf

[82] Eurostat. Unemployment rate by sex and age groups-annual average, % http://appsso.eurostat.ec.europa.eu/nui/show.do?dataset=une_rt_a&lang=en

[83] The World Bank. GDP growth (%) http://data.worldbank.org/indicator/NY.GDP.MKTP.KD.ZG

[84] Van der Bracht K, Van de Putte B (2014). Homonegativity among first and second generation migrants in Europe: the interplay of time trends, origin, destination and religion. Soc Sci Res..

[85] Stegmueller D (2013). How many countries for multilevel modeling? A comparison of frequentist and Bayesian approaches. Am J Polit Sci..

[86] Fairbrother M (2014). Two multilevel modeling techniques for analyzing comparative longitudinal survey datasets. Political Science Research and Methods..

[87] Jaccard J (2001). Interaction effects in logistic regression.

[88] Mood C (2010). Logistic regression: why we cannot do what we think we can do, and what we can do about it. Eur Sociol Rev..

[89] Utzet M, Moncada S, Molinero E, Llorens C, Moreno N, Navarro A (2014). The changing patterns of psychosocial exposures at work in the South of Europe: Spain as a labor market laboratory. Am J Ind Med..

[90] Clark A, Knabe A, Ratzel S (2010). Boon or bane? Others’ unemployment, well-being and job insecurity. Labour Econ..

[91] Clark AE (2003). Unemployment as a social norm: psychological evidence from panel data. J Labor Econ..

[92] Eurofound (2012). Third European quality of life survey-quality of life in Europe: impacts of the crisis.

[93] De Vogli R. Financial crisis, austerity, and health in Europe. Lancet. 2013;391 10.1016/S0140-6736(13)61662-1 23911369

[94] de Belvis AG, Ferre F, Specchia ML, Valerio L, Fattore G, Ricciardi W (2012). The financial crisis in Italy: implications for the healthcare sector. Health Policy..

[95] Van Doorslaer E, Jones AM (2004). Income-related inequality in health and health care in the European Union. Health Econ..

[96] Vasiliadis HM, Tempier R, Lesage A, Kates N (2009). General practice and mental health care: determinants of outpatient service use. Can J Psychiat..

[97] Buffel V, Colman E, Dereuddre R, Bracke P (2014). The use of mental health care, psychotropic drugs and social services by divorced people: does informal support matter? European Journal of Social Work.

[98] Cooper B (2011). Economic recession and mental health: an overview. Neuropsychiatry..

[99] Vuori J, Silvonen J, Vinokur AD, Price RH (2002). The tyohon Job search program in Finland: benefits for the unemployed with risk of depression or discouragement. J Occup Health Psychol..

[100] Virtanen P (1993). Unemployment, re-employment and the use of primary health care services. Scand J Prim Health Care..

[101] Gerdtham UG, Johannesson M (2005). Business cycles and mortality: results from Swedish microdata. Soc Sci Med..

