# Supplementary material for: Employment status and mental health care use in times of economic contraction: a repeated cross-sectional study in Europe, using a three-level model
Source: Int J Equity Health. 2015 Mar 11;14:29. doi: 10.1186/s12939-015-0153-3 (PMC4367872; doi:10.1186/s12939-015-0153-3)
Supplement: Additional file 1: Table S1. — Percentage of cases with missing values on the variables and the final sample size of women and men per country per period. Source: Eurobarometer wave 58.2 (2002), wave 64.4 (2005/2006) and wave 73.2 (2010). [file 12939_2015_153_MOESM1_ESM.doc]

Additional file 1: Table S1. Percentage of cases with missing values on the variables and the final sample size of women and men per country per period

|  | **Women (N=28 014)** | | | | | | **Men (N=22 978)** | | | | | |
| --- | --- | --- | --- | --- | --- | --- | --- | --- | --- | --- | --- | --- |
|  | **2002** | | **2005/2006** | | **2010** | | **2002** | | **2005/2006** | | **2010** | |
|  | **Missings** | **Final** | **Missings** | **Final** | **Missings** | **Final** | **Missings** | **Final** | **Missings** | **Final** | **Missings** | **Final** |
| Country | **(%)** | **N** | **(%)** | **N** | **(%)** | **N** | **(%)** | **N** | **(%)** | **N** | **(%)** | **N** |
| Belgium | 0.71 | 419 | 1.46 | 404 | 8.17 | 371 | 1.68 | 409 | 2.58 | 340 | 6.99 | 359 |
| Denmark | 1.04 | 381 | 0.80 | 373 | 1.60 | 370 | 0.51 | 390 | 7.29 | 356 | 0.29 | 348 |
| Germany | 0.76 | 781 | 0.49 | 609 | 1.19 | 581 | 0.82 | 726 | 0.19 | 515 | 0.95 | 523 |
| Greece | 0.00 | 401 | 1.05 | 469 | 7.16 | 363 | 0.00 | 371 | 0.35 | 283 | 0.82 | 363 |
| Spain | 0.26 | 386 | 0.49 | 408 | 1.57 | 375 | 0.00 | 364 | 0.74 | 267 | 0.28 | 355 |
| France | 1.40 | 424 | 0.24 | 424 | 2.14 | 412 | 1.01 | 392 | 1.48 | 333 | 1.44 | 343 |
| Ireland | 1.01 | 392 | 4.80 | 456 | 4.43 | 453 | 0.79 | 378 | 6.42 | 306 | 1.45 | 340 |
| Italy | 0.49 | 403 | 1.17 | 508 | 3.29 | 412 | 0.78 | 384 | 2.92 | 333 | 3.52 | 356 |
| Luxembourg | 0.78 | 254 | 0.45 | 219 | 2.43 | 201 | 0.00 | 212 | 3.47 | 139 | 1.22 | 162 |
| Netherlands | 1.12 | 441 | 1.13 | 438 | 2.04 | 385 | 1.90 | 414 | 0.92 | 432 | 2.66 | 366 |
| Portugal | 0.25 | 401 | 2.49 | 352 | 3.47 | 389 | 0.29 | 339 | 2.39 | 245 | 5.29 | 340 |
| United Kingdom | 1.07 | 648 | 1.15 | 517 | 3.31 | 467 | 2.86 | 374 | 1.94 | 404 | 0.00 | 404 |
| Austria | 1.45 | 476 | 1.13 | 436 | 2.69 | 434 | 3.12 | 311 | 4.49 | 383 | 3.00 | 356 |
| Sweden | 1.45 | 407 | 1.36 | 362 | 2.62 | 334 | 1.41 | 350 | 1.02 | 387 | 1.54 | 319 |
| Finland | 1.86 | 423 | 0.73 | 408 | 1.17 | 337 | 1.22 | 325 | 3.95 | 292 | 2.20 | 311 |
| Republic of Cyprus |  |  | 0.00 | 226 | 2.45 | 199 |  |  | 0.00 | 165 | 0.00 | 183 |
| Czech Republic |  |  | 0.45 | 443 | 3.20 | 424 |  |  | 1.34 | 369 | 3.98 | 362 |
| Estonia |  |  | 0.49 | 409 | 3.16 | 398 |  |  | 0.89 | 223 | 1.26 | 314 |
| Hungary |  |  | 1.76 | 447 | 3.81 | 404 |  |  | 0.72 | 275 | 1.98 | 346 |
| Latvia |  |  | 0.22 | 451 | 7.20 | 361 |  |  | 0.66 | 301 | 9.04 | 332 |
| Lithuania |  |  | 0.24 | 412 | 4.00 | 384 |  |  | 1.53 | 258 | 3.89 | 321 |
| Malta |  |  | 0.79 | 251 | 5.63 | 218 |  |  | 0.00 | 112 | 3.88 | 124 |
| Poland |  |  | 0.74 | 400 | 3.08 | 440 |  |  | 2.29 | 341 | 2.62 | 297 |
| Slovakia |  |  | 2.91 | 467 | 1.83 | 429 |  |  | 3.41 | 312 | 1.45 | 407 |
| Slovenia |  |  | 3.37 | 402 | 7.82 | 377 |  |  | 5.11 | 334 | 8.18 | 303 |
| Bulgaria |  |  | 0.80 | 373 | 2.71 | 430 |  |  | 3.54 | 354 | 2.67 | 292 |
| Romania |  |  | 4.83 | 394 | 11.06 | 378 |  |  | 11.97 | 309 | 9.16 | 357 |
| Total | 0.93 | 6637 | 1.36 | 11058 | 3.82 | 10326 | 1.10 | 5739 | 2.80 | 8368 | 3.13 | 8871 |
