# Supplementary material for: Employment status and mental health care use in times of economic contraction: a repeated cross-sectional study in Europe, using a three-level model
Source: Int J Equity Health. 2015 Mar 11;14:29. doi: 10.1186/s12939-015-0153-3 (PMC4367872; doi:10.1186/s12939-015-0153-3)
Supplement: Additional file 2: Table S2. — Description of the sample: the individual variables by period and gender. and their % missing values. Source: Eurobarometer wave 58.2 (2002), wave 64.4 (2005/2006) and wave 73.2 (2010). [file 12939_2015_153_MOESM2_ESM.doc]

Additional file 2: Table S2 Description of the sample: the individual variables by period and gender. and their % missing values.

|  | **% missing values** | | **2002** | | | | **2005/2006** | | | | **2010** | | | | **Total** | | | |
| --- | --- | --- | --- | --- | --- | --- | --- | --- | --- | --- | --- | --- | --- | --- | --- | --- | --- | --- |
|  | **Women** | **Men** | **Women** | | **Men** | | **Women** | | **Men** | | **Women** | | **Men** | | **Women** | | **Men** | |
| **Mental health** (x̅, SD) | 0.3 | 0.3 | 3.7 | 0.8 | 3.8 | 0.8 | 3.8 | 0.8 | 3.9 | 0.7 | 3.6 | 0.7 | 3.8 | 0.7 | 3.7 | 0.8 | 3.8 | 0.8 |
| **Mental health care use** (%) |  |  |  |  |  |  |  |  |  |  |  |  |  |  |  |  |  |  |
| GP consultations | 0.1 | 0.1 | 6.4 |  | 4.1 |  | 10.9 |  | 7.6 |  | 12.4 |  | 9.0 |  | 10.4 |  | 7.3 |  |
| Psychiatrist consultations | 0.1 | 0.2 | 1.7 |  | 1.4 |  | 2.3 |  | 1.6 |  | 1.8 |  | 1.5 |  | 2.0 |  | 1.5 |  |
| **Employment status** (%) | 1.7 | 1.3 |  |  |  |  |  |  |  |  |  |  |  |  |  |  |  |  |
| Employed |  |  | 55.8 |  | 74.1 |  | 54.4 |  | 72.5 |  | 54.7 |  | 67.9 |  | 54.8 |  | 71.1 |  |
| Non-employed |  |  | 37.8 |  | 19.9 |  | 37.8 |  | 20.2 |  | 35.3 |  | 20.5 |  | 36.9 |  | 20.3 |  |
| Unemployed |  |  | 6.3 |  | 6.0 |  | 7.8 |  | 7.3 |  | 10.0 |  | 11.6 |  | 8.3 |  | 8.7 |  |
| **Age** (x̅, SD) | 0.0 | 0.0 | 41.3 | 12.8 | 41.7 | 13.1 | 43.4 | 12.9 | 43.0 | 13.2 | 43.6 | 12.9 | 43.3 | 13.1 | 43.0 | 12.9 | 42.8 | 13.1 |
| **Education** (%) | 0.2 | 0.1 |  |  |  |  |  |  |  |  |  |  |  |  |  |  |  |  |
| Low |  |  | 36.0 |  | 33.3 |  | 27.9 |  | 24.5 |  | 23.3 |  | 21.2 |  | 28.1 |  | 25.4 |  |
| Middle |  |  | 30.0 |  | 30.1 |  | 38.0 |  | 39.1 |  | 39.5 |  | 41.2 |  | 36.7 |  | 37.2 |  |
| High |  |  | 34.0 |  | 36.6 |  | 32.9 |  | 34.8 |  | 31.4 |  | 31.4 |  | 32.6 |  | 33.9 |  |
| **Marital status** (%) | 0.9 | 1.1 |  |  |  |  |  |  |  |  |  |  |  |  |  |  |  |  |
| Married/ legally cohabiting |  |  | 55.8 |  | 52.8 |  | 67.2 |  | 69.0 |  | 68.5 |  | 69.7 |  | 65.0 |  | 65.3 |  |
| Single |  |  | 29.1 |  | 38.7 |  | 14.5 |  | 21.8 |  | 16.5 |  | 22.1 |  | 18.7 |  | 26.1 |  |
| Divorced |  |  | 10.6 |  | 7.1 |  | 11.2 |  | 7.4 |  | 9.9 |  | 6.7 |  | 10.6 |  | 7.1 |  |
| Widow(er) |  |  | 4.6 |  | 1.4 |  | 7.0 |  | 1.7 |  | 5.1 |  | 1.5 |  | 5.7 |  | 1.6 |  |
| **Degree of urbanization** (%) | 0.2 | 0.2 |  |  |  |  |  |  |  |  |  |  |  |  |  |  |  |  |
| Rural |  |  | 35.5 |  | 37.3 |  | 36.0 |  | 35.9 |  | 36.3 |  | 35.7 |  | 36.0 |  | 36.2 |  |
| Small urban |  |  | 31.5 |  | 29.4 |  | 36.5 |  | 36.5 |  | 35.7 |  | 34.6 |  | 35.0 |  | 34.0 |  |
| Large urban |  |  | 33.0 |  | 33.3 |  | 27.5 |  | 27.6 |  | 28.0 |  | 29.7 |  | 29.0 |  | 29.8 |  |
