# Supplementary material for: Employment status and mental health care use in times of economic contraction: a repeated cross-sectional study in Europe, using a three-level model
Source: Int J Equity Health. 2015 Mar 11;14:29. doi: 10.1186/s12939-015-0153-3 (PMC4367872; doi:10.1186/s12939-015-0153-3)
Supplement: Additional file 3: Table S3. — Description of the external data: Real GDP growth rate and unemployment rate of women and men by period and country. Source: World Bank for real GDP growth rates; Eurostat for the unemployment rates (2001, 2004 and 2009). [file 12939_2015_153_MOESM3_ESM.doc]

Additional file 3: Table S3 Description of the external data: Real GDP growth rate and unemployment rate of women and men by period and country.

|  | **Real GDP growth rate** | | | **Unemployment rate of women** | | | **Unemployment rate of men** | | |
| --- | --- | --- | --- | --- | --- | --- | --- | --- | --- |
|  | **2001/2002** | **2004/2005** | **2009/2010** | **2001/2002** | **2004/2005** | **2009/2010** | **2001/2002** | **2004/2005** | **2009/2010** |
| Belgium | 0.9 | 3.4 | −2.6 | 7.5 | 9.5 | 8.1 | 5.9 | 7.5 | 7.8 |
| Denmark | 0.8 | 2.6 | −5.1 | 5.5 | 6.0 | 5.3 | 4.1 | 5.1 | 6.6 |
| Germany | 1.7 | 1.2 | −5.6 | 7.9 | 10.1 | 7.2 | 7.7 | 10.6 | 8.0 |
| Greece | 3.7 | 5.0 | −4.4 | 16.1 | 16.3 | 13.3 | 7.2 | 6.7 | 7.0 |
| Spain | 4.0 | 3.2 | −3.6 | 15.2 | 14.8 | 18.1 | 7.5 | 8.3 | 17.7 |
| France | 2.0 | 2.8 | −2.9 | 7.8 | 9.7 | 9.2 | 7.9 | 8.2 | 9.0 |
| Ireland | 5.3 | 4.6 | −6.4 | 3.8 | 4.0 | 8.2 | 4.1 | 4.8 | 15.0 |
| Italy | 1.8 | 1.6 | −5.5 | 12.1 | 10.5 | 9.3 | 6.9 | 6.4 | 6.8 |
| Luxembourg | 2.0 | 4.9 | −5.3 | 2.4 | 6.8 | 5.9 | 1.6 | 3.6 | 4.5 |
| Netherlands | 1.6 | 1.9 | −3.3 | 3.2 | 5.3 | 3.8 | 2.1 | 4.9 | 3.7 |
| Portugal | 1.9 | 1.8 | −3.0 | 5.1 | 7.7 | 10.3 | 5.2 | 7.9 | 11.0 |
| United Kingdom | 2.7 | 2.5 | −4.3 | 4.4 | 4.3 | 6.4 | 5.5 | 5.1 | 8.5 |
| Austria | 1.4 | 2.7 | −3.8 | 4.2 | 5.4 | 4.6 | 3.1 | 4.5 | 5.0 |
| Sweden | 1.6 | 4.3 | −5.2 | 5.6 | 7.1 | 8.0 | 6.1 | 7.6 | 8.6 |
| Finland | 2.6 | 3.9 | −8.3 | 9.7 | 8.9 | 7.6 | 8.6 | 8.7 | 8.9 |
| Republic of Cyprus |  | 4.2 | −1.7 |  | 6.0 | 5.5 |  | 3.5 | 5.3 |
| Czech Republic |  | 4.9 | −4.8 |  | 9.9 | 7.7 |  | 7.0 | 5.9 |
| Estonia |  | 6.5 | −14.7 |  | 9.1 | 10.3 |  | 11.1 | 16.7 |
| Hungary |  | 4.8 | −6.6 |  | 6.1 | 9.7 |  | 6.1 | 10.3 |
| Latvia |  | 8.7 | −18.0 |  | 12.0 | 14.1 |  | 11.5 | 20.9 |
| Lithuania |  | 7.4 | −14.7 |  | 11.3 | 10.5 |  | 10.5 | 17.1 |
| Malta |  | −0.5 | −2.8 |  | 9.0 | 7.6 |  | 6.4 | 6.5 |
| Poland |  | 5.1 | 2.6 |  | 20.1 | 8.6 |  | 18.3 | 7.8 |
| Slovakia |  | 5.2 | −5.3 |  | 19.3 | 12.9 |  | 17.5 | 11.5 |
| Slovenia |  | 4.4 | −7.8 |  | 6.9 | 5.8 |  | 5.9 | 5.9 |
| Bulgaria |  | 6.6 | −5.0 |  | 11.6 | 6.7 |  | 12.5 | 6.9 |
| Romania |  | 9.1 | −6.8 |  | 6.8 | 5.4 |  | 8.9 | 7.3 |
